# Retraction: Sequestration of chromium(vi) and nickel(ii) heavy metals from unhygienic water *via* sustainable and innovative magnetic nanotechnology

**DOI:** 10.1039/d4na90109f

**Published:** 2024-10-15

**Authors:** Noor Zulfiqar, Monireh Shariatipour, Fawad Inam

**Affiliations:** a Department of Chemistry, Faculty of Science, University of Agriculture Faisalabad Pakistan chemistnoor94@gmail.com 2018ag3898@uaf.edu.pk; b Department of Chemistry, Faculty of Science, Tarbiat Modares University Tehran Iran; c School of Architecture, Computing and Engineering, University of East London EB 1.102 Docklands Campus, University Way London E16 2RD UK; d Executive Principal Office, Oxford Business College 23-38 Hythe Bridge Street Oxford OX1 2EP UK

## Abstract

Retraction of ‘Sequestration of chromium(vi) and nickel(ii) heavy metals from unhygienic water *via* sustainable and innovative magnetic nanotechnology’ by Noor Zulfiqar *et al.*, *Nanoscale Adv.*, 2024, **6**, 287–301, https://doi.org/10.1039/D3NA00923H.

The Royal Society of Chemistry hereby wholly retracts this *Nanoscale Advances* article due to concerns with the reliability of the characterisation data in the published article.

The X-ray powder diffraction data in Fig. 2 contains anomalies and unusual peak shapes. Subsequent recharacterization of the material by the authors gave significantly different diffraction data. Additionally, the particle sizes were stated to be ∼8 and ∼9 nm in the original paper but subsequent recharacterization resulted in particles sizes of ∼18 nm. An expert has reviewed the data and concluded that the material characterisation is inconsistent, and the original conclusions of the work are therefore not reliable.

The authors do not agree with this retraction.

Jeremy Allen, Executive Editor, *Nanoscale Advances*

8th October 2024

